# Squamous cell carcinoma arising from giant porokeratosis and rare postoperative recurrence and metastasis: A case report

**DOI:** 10.1097/MD.0000000000018697

**Published:** 2020-01-10

**Authors:** Fang Zhang, Wenzhe Bai, Shuna Sun, Na Li, Xiaojie Zhang

**Affiliations:** a First Clinical Medical College, Shandong University of Traditional Chinese Medicine; b Department of Dermatology, The Affiliated Hospital of Shandong University of Traditional Chinese Medicine, Shandong Provincial Hospital of Traditional Chinese Medicine, Jinan, Shandong, China.

**Keywords:** metastasis, porokeratosis, squamous cell carcinoma

## Abstract

**Rationale:**

Giant porokeratosis is considered to be a variant of porokeratosis of Mibelli (PM) by some medical scholars. Porokeratosis can develop into several epidermal malignant tumors, such as Bowen disease and basal cell carcinoma, among which squamous cell carcinoma (SCC) is the most common.

**Patient concerns:**

The patient was a 53-year-old man who was admitted to our hospital due to postoperative recurrence and metastasis as SCC arising from giant PM in his left leg and foot.

**Diagnoses:**

The pathological results are porokeratosis and well-differentiated squamous cell carcinoma. Positron emission tomography and computed tomography results show the local recurrence of the tumor with multiple lymph node metastasis.

**Interventions:**

This patient was transferred to orthopedic surgery for amputation of the middle and lower left thigh.

**Outcomes:**

Follow-up for 3 months has shown no recurrence after the surgery.

**Lessons:**

This report reminds us to pay close attention to the likelihood of giant porokeratosis. The physicians should explore all clinical possibilities to avoid misdiagnosis of this rare disease.

Although the recurrence rate of SCC arising from giant PM is very low, the surgical resection region should be expanded appropriately such as the en-block resection.

## 1 Introduction

Porokeratosis is a rare form of keratinization disease, which was first described by Isidor Neuman in 1875 and first named by Italian scholar Mibelli in 1893 because of the involvement of eccrine ostia in his case.^[1]^ In addition to classic porokeratosis of Mibelli (PM), Porokeratosis also can be classified as: disseminated superficial actinic porokeratosis, disseminated porokeratosis, linear porokeratosis, porokeratosis punctate palmaris et plantaris, and porokeratosis palmaris et plantaris disseminate.^[2]^ PM frequently appears in childhood with a higher morbidity rate in males than female (2.17:1). Histologically, the cornoid lamella is the hallmark of all porokeratosis variants. Moreover, the granular cell layer decreased or disappeared below the column of parakeratotic cells. Chronic inflammatory cells infiltrate into the perivascular space of the dermal superficial layer. Clinical reports of malignant tumors secondary to porokeratosis have increased steadily increased, with squamous cell carcinoma (SCC) being the most common.^[3]^

## 2 Case report

A 53-year-old man presented with a history of an erythematous irregular plaque on the left leg and foot since birth that had gradually increased in size with mild pruritus. While seeking medical treatment at many different hospitals, his disease was always misdiagnosed as psoriasis or epidermal nevus. Oral antihistamines and locally potent steroids had no apparent effect. The patient finally gave up on medical treatment for financial reasons.

**Figure 1:**
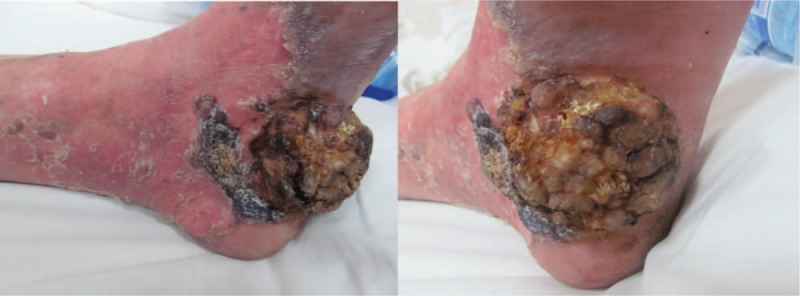
Ulcerated cauliflower-like tumor within giant porokeratosis on the medial side of the left heel 7 yr ago.

**Figure 2:**
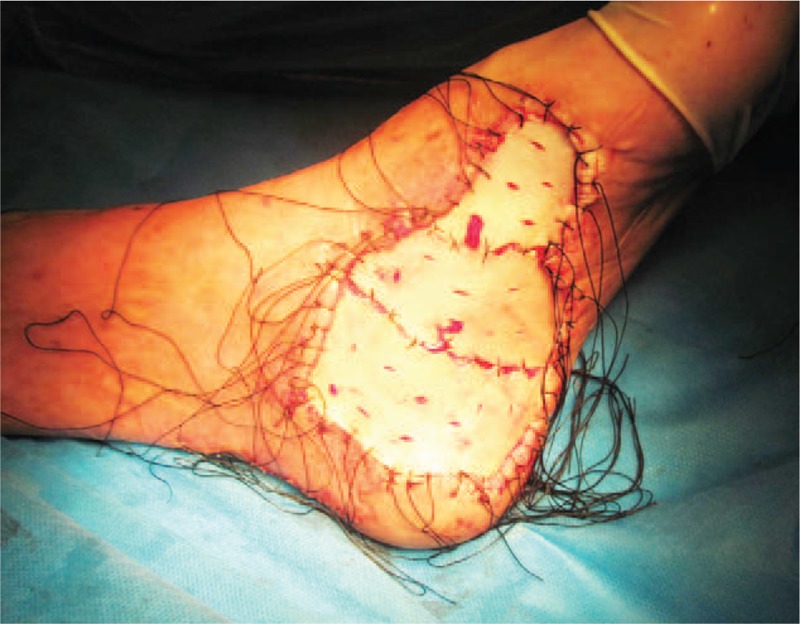
The patient received skin grafting after surgical local excision 7 yr ago.

**Figure 3:**
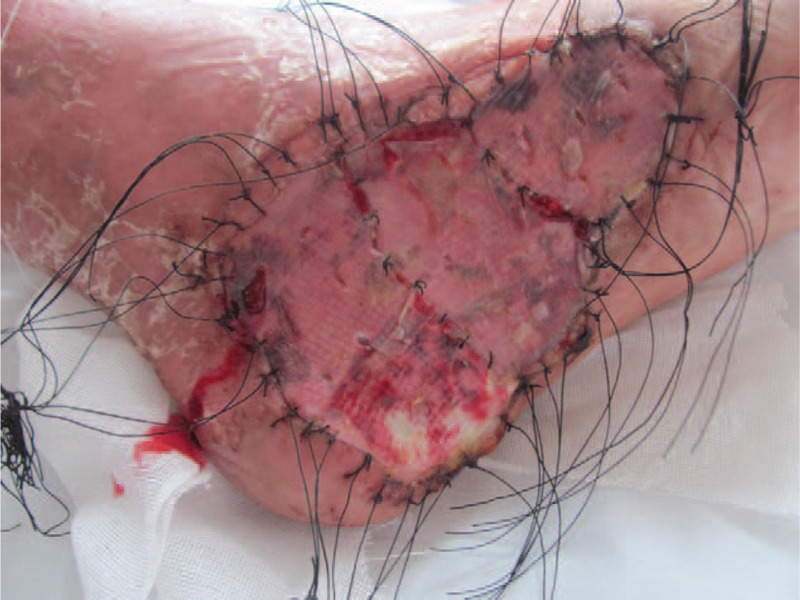
Survival condition of grafted skin 7 yr ago.

Seven years ago, a red papule developed in the lesion and continued to grow until it became an evident tumor. Physical examination revealed a 5 × 5 × 4 cm ulcerated cauliflower-like tumor on the medial side of the left heel, accompanied by some purulent discharge. The discharge was located in a scaly hyperkeratotic erythematous plaque with central atrophy surrounded by a well-defined, slightly raised, hyperkeratotic wall (Fig. 1). There was no family history of similar lesions. The pathological results were SCC and porokeratosis. The diagnosis of SCC arising from giant porokeratosis was reached. The patient received skin grafting after surgical local excision (Figs. 2 and 3), but the patient refused treatment for the giant porokeratosis. The patient did not follow advice of the medical team to attend regular follow-up.

**Figure 4:**
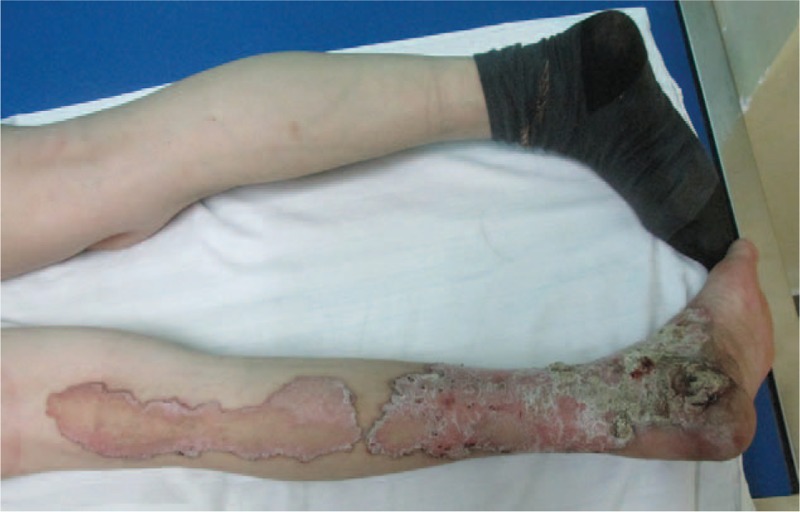
The image of patient skin: the scaly erythematous plaque with raised keratotic border along with atrophy and hair loss at the center of the lesion. There is a well-defined tumor with a size of 5 × 4 cm on the surface of the lesion.

**Figure 5:**
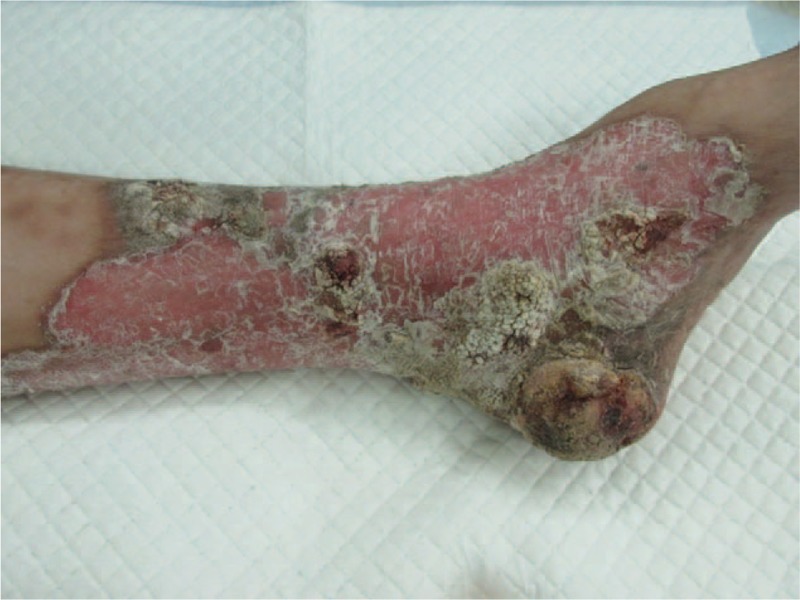
Large, ulcerated tumor within giant porokeratosis.

**Figure 6:**
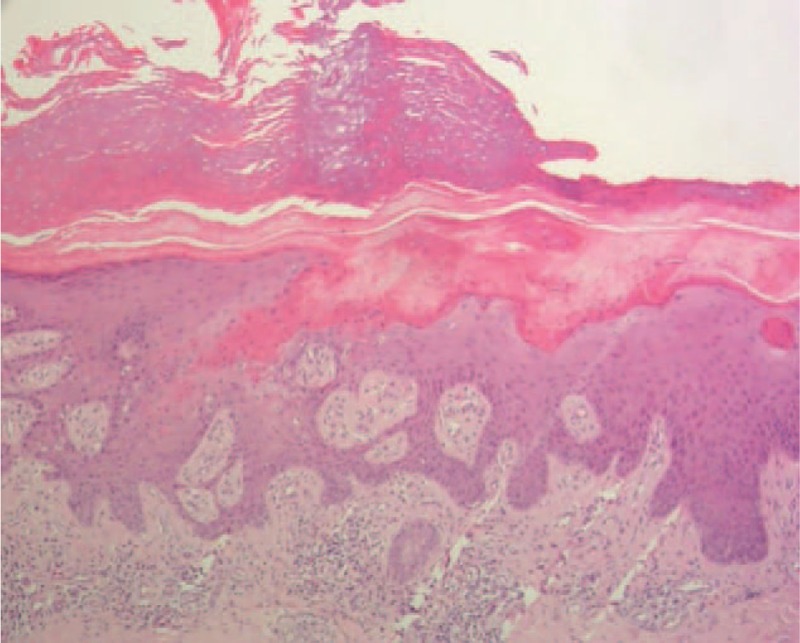
Parakeratinized column of parakeratinized cells in the stratum corneum, reduced or unobservable inferion granular layer, and scattered dyskeratotic cells in the prickle cell layer.

**Figure 7:**
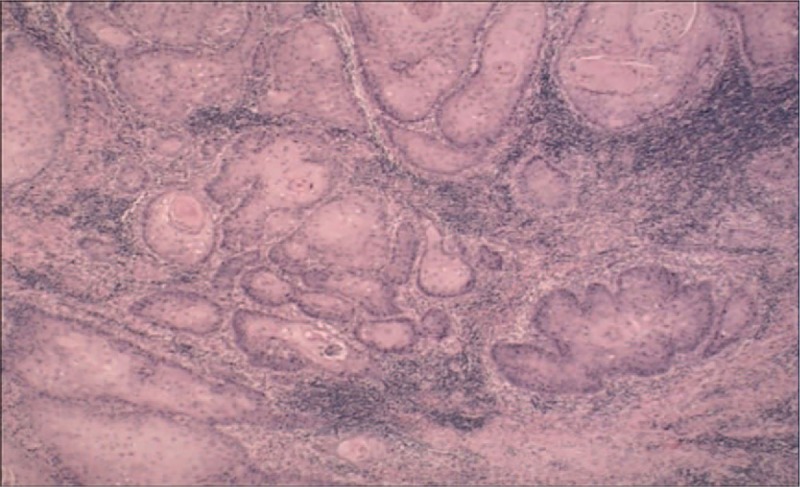
Histologic findings from tumor biopsy specimen of tumor showed characteristic features of squamous cell carcinoma (H&E; original magnification ×100).

New malignant cells were found in the same region where the first tumor was discovered and the clinical manifestations were also almost the same as previously (Figs. 4 and 5). The area of the porokeratosis did not change. The total size of porokeratosis was about 50% of the skin area of the left calf and foot. The size of the new tumor was about 4 × 3 × 2 cm. The movement of the left ankle joint was normal and blood flow at the end and the skin felt normal. Routine blood tests, biochemical examinations, and chest X-ray were all normal, and no evidence of immunosuppression was detected. The remaining cutaneous and systemic examinations were all within normal conditions. Biopsy of the tumor showed characteristic features of porokeratosis and well-differentiated SCC (Figs. 6 and 7). Positron emission tomography and computed tomography (CT) showed that 18F-fluorodeoxyglucose metabolism in the left leg was increased and the local recurrence of the tumor with multiple lymph node metastasis was considered. The patient was referred to the department of bone oncology for amputation of the middle and lower left thigh. The operation was successful and the incision healed well. The patient was regularly followed up in the department of bone oncology. In the recent follow-up, the patient was able to walk with artificial limb, and color Doppler ultrasound showed no significant enlargement of the left inguinal lymph nodes. There was no abnormality in pulmonary CT

## 3 Discussion

PM is a disorder of epidermal keratinization characterized by annular plaques with an atrophic center surrounded by a keratotic wall. PM is clinically similar to linear moss, plaque psoriasis, and inflammatory linear verrucous epidermal nevus. As there is a lack of understanding about this disease, it is often misdiagnosed or undiagnosed. In addition to biopsy, dermoscopy is a rapid technique that can aid in the accurate diagnosis of PM and exclude disease that may clinically mimic PM.^[4]^

It is estimated that 7.5% to 11% of patients with porokeratosis have metastasis, SCC being the most common.^[5]^ In patients with the following risk factors, porokeratosis is more likely to develop malignant lesions:

(1) skin lesions occur on the extremities with a large area;
(2) a long disease course;
(3) older persons;
(4) sun exposure;
(5) immunosuppression.^[6]^

In the present study, surgical excision is a viable and effective treatment strategy for SCC causing by porokeratosis, while the recurrence rate is very low. There is a lack of unified and effective treatment for giant porokeratosis, because its mechanism has not been elucidated completely. In addition to surgery, treatment methods include:

(1) Physiotherapy: fractional photothermolysis, laser therapy, dermabrasion, cryotherapy, and photodynamic therapy;
(2) Topical treatment: retinoids, diclofenac gel, fluorouracil cream, imiquimod cream, vitamin D3 analogs;
(3) Systemic treatment: retinoids.^[7]^

External stimulation of the lesions should be avoided, like from ultraviolet rays, abrasion or pressure.

Malignant metastasis of SCC caused by giant plaque porokeratosis, as observed in this case, is extremely rare.^[8]^ To our knowledge, this is the most severe case of postoperative recurrence and metastasis of malignant SCC caused by giant porokeratosis ever reported. Therefore, close attention is required for the diagnosis and treatment of porokeratosis to reduce risk of cancer. In addition, regular follow-up is indispensable for the prevention and treatment of recurrence.

## Acknowledgment

We thank Professor Yukun Wang of the Department of Dermatology, Qilu Hospital, Shandong University for pathological guidance.

## Author contributions

**Conceptualization:** Xiaojie Zhang.

**Data curation:** Fang Zhang.

**Investigation:** Wenzhe Bai.

**Methodology:** Fang Zhang, Wenzhe Bai, Shuna Sun, Na Li.

**Writing – original draft:** Fang Zhang.

**Writing – review and editing:** Xiaojie Zhang.
